# Case Report: Clinical and genetic analysis of a Bietti crystalline dystrophy family with multisite crystalline deposits and a phenotype of macular hole combined with retinoschisis

**DOI:** 10.3389/fmed.2025.1664126

**Published:** 2026-01-15

**Authors:** Cheng-yao Zheng, Hong Chen, Yu-ying Jiang, Hong Lu

**Affiliations:** Eye Institute, Affiliated Hospital of Nantong University, Medical School of Nantong University, Nantong, Jiangsu, China

**Keywords:** BCD, Bietti crystalline dystrophy, CYP4V2, retinal degeneration, retinoschisis

## Abstract

**Purpose:**

To identify and characterize the genetic mutation responsible for Bietti crystalline dystrophy (BCD) in a Chinese consanguineous family and to describe the associated clinical phenotypes.

**Methods:**

Comprehensive genetic screening was performed on a Chinese consanguineous family using targeted next-generation sequencing (NGS) of 1,276 ophthalmology-related genes, followed by confirmation with Sanger sequencing. All family members underwent detailed ophthalmologic examinations.

**Results:**

We investigated a consanguineous Chinese family affected by Bietti crystalline dystrophy (BCD). The proband, a 46-year-old woman, reported progressive bilateral visual decline and nyctalopia over a span of two decades. Her younger sister exhibited similar symptoms. Ophthalmic examinations revealed crystalline deposits involving the cornea, retina, and lens. Notably, in addition to the previously reported crystal-like deposits on the inner surface of the anterior lens capsule, our cases also demonstrated crystals on the anterior surface of the capsule and within the lens cortex, with the proband showing more extensive involvement than her sister. This novel pattern of anterior capsular surface involvement provides a new perspective on the possible pathways of crystal deposition in BCD. Optical coherence tomography (OCT) revealed cystoid macular edema (CME) in the proband, whereas her sister presented with a rare combination of a full-thickness macular hole (MH) and retinoschisis—an OCT phenotype not previously reported in BCD. Next-generation sequencing (NGS) identified a homozygous splice-site mutation (c.1091-2A > G) in CYP4V2 in the proband, which was confirmed in her sister by Sanger sequencing. Other family members were found to be heterozygous carriers. Genotype–phenotype co-segregation supported the pathogenicity of this variant.

**Conclusion:**

The homozygous CYP4V2 c.1091-2A > G mutation was identified as the disease-causing variant in this family. This study broadens the phenotypic spectrum of BCD by highlighting multisite crystalline deposits and rare macular structural changes. These findings suggest that crystal deposition in BCD affects multiple ocular tissues and involves complex pathological mechanisms. Combined multimodal imaging and genetic testing can enhance the recognition and diagnosis of BCD.

## Introduction

Bietti crystalline dystrophy (BCD; OMIM 210370) is a rare autosomal recessive retinal dystrophy first described by Italian ophthalmologist G. B. Bietti in 1937 (1). BCD is characterized by numerous shimmering yellow-white crystalline deposits at the posterior pole of the retina associated with, and in some cases, similar crystalline deposits are also observed at the corneoscleral limbus. BCD is also accompanied by RPE atrophy and pigment clumping (2). The prevalence of Bietti Crystalline Dystrophy (BCD) varies significantly across regions and ethnic groups, ranging from extremely rare (1,4,500,000 in Spain and 1,500,000 in Israel) to relatively common (1,24,000 in China) (3).

A 2004 study identified that mutations in the CYP4V2 gene are responsible for the development of BCD (4). The CYP4V2 gene, a member of the cytochrome P450 hemethiolate protein superfamily, spans 19 kb with 11 exons and encodes fatty acid *ω*-hydroxylase that metabolizes both saturated and unsaturated fatty acids (5). Among the 1,171 currently known CYP4V2 mutations, 156 are classified as pathogenic, with missense mutations being the most common type and splice-site mutations the rarest (3). The three most prevalent CYP4V2 mutations are: c.802-8_810del17insGC (Exon7del), c.992A > C (p.H331P), c.1091-2A > G (Exon9del) (6). This study presents clinical characterization and next-generation sequencing (NGS) analysis of a consanguineous Chinese pedigree exhibiting autosomal recessive Bietti crystalline dystrophy (BCD), resulting from a second cousin marriage.

## Methods

### Ethical approval

This study was conducted at the Affiliated Hospital of Nantong University (Nantong, China). The study was performed in accordance with the tenets of the Declaration of Helsink. Prior to enrollment, the study was approved by the institutional ethics committee (approval number: 2022-L100), and written informed consent was obtained from all participants.

### Clinical studies

Seven family members all underwent ophthalmologic examinations, including best-corrected visual acuity (BCVA), intraocular pressure (IOP), slit lamp biomicroscopy, fundus examination and photography, and optical coherence tomography (OCT) were performed. The clinical data were assessed by ophthalmologists at Affiliated Hospital of Nantong University.

### Molecular genetic studies

#### DNA isolation

Two milliliters of peripheral blood were collected from each of the seven individuals with EDTA anticoagulant. Genomic DNA was obtained from their peripheral blood by QIA amp DNA Blood Mini Kit (Qiagen, Hilden, Germany). DNA quality control was performed using the Nanodrop 2000 spectrophotometer (Thermo Fisher Scientific, DE, USA).

#### DNA library preparation

DNA library preparation was performed using a standard library construction kit (MyGenostics, Beijing, China). The constructed libraries were amplified by PCR, and the PCR products were purified using Ampure XP beads (Beckman Coulter, Brea, CA, USA). The quality control of the constructed libraries was performed using the Nanodrop 2000 spectrophotometer and agarose gel electrophoresis was conducted to verify the size distribution of the library fragments.

#### Target region capture

Targeted regions were enriched using the GenCap liquid-phase capture kit (MyGenostics, Beijing, China), which includes probes for 1,276 genes associated with the disease phenotype. The biotin-labeled probes were hybridized with the library DNA. The biotin-labeled probes were captured by streptavidin-coated magnetic beads through covalent binding, allowing for the specific isolation of target genes. The beads bound to the target genes were then separated using a magnetic stand, and the target genes were eluted and purified to achieve enrichment. The captured products were subjected to quality control again.

#### Next-generation sequencing (NGS)

The prepared library is immobilized on a high-density sequencing chip (Flowcell), The NextSeq 500 sequencing system (Illumina, Inc.) subsequently performs automated cycles of sequencing-by-synthesis (SBS) and fluorescence imaging, enabling high-throughput and accurate determination of the complete nucleic acid sequence.

#### Mutation identification

Cutadapt (v.1.16) was employed to trim adaptor sequences, filter out low-quality reads, discard reads containing excessive Ns, and remove short reads (<40 bp) to ensure high-quality data for analysis. The preprocessed sequencing reads were aligned to the human reference genome (hg19) using the Burrows-Wheeler Aligner (BWA, v.0.7.10). PCR duplicates were removed or marked using the MarkDuplicates tool from the Genome Analysis Toolkit (GATK, v.4.0.8.1). This step eliminated artifacts introduced during library amplification, thereby reducing false-positive variant calls. Base quality scores were recalibrated using the BaseRecalibrator tool from GATK and the recalibrated data were then output using the ApplyBQSR tool from GATK for subsequent variant detection analysis. Single nucleotide polymorphisms (SNPs) and insertions/deletions (INDELs) were identified using the HaplotypeCaller tool from GATK.

### Data analysis

The identified SNPs and INDELs were functionally annotated using the ANNOVAR software (v.2018.04.16) and custom scripts, integrating information from multiple databases, including dbSNP, ClinVar, Human Gene Mutation Database, 1,000 Genomes project and Exome Sequencing Project (ESP). Variant filtering and pathogenicity analysis were performed following the American College of Medical Genetics and Genomics (ACMG) guidelines.

### Sanger sequencing

After candidate causative mutations were identified, Sanger sequencing was performed to validate the mutations. DNA extraction and quality control were conducted following the same protocols as previously described. PCR amplification was set up using the Beckman automated workstation (Beckman Coulter, Brea, CA, USA), and thermal cycling was performed with specific primers (forward: 5′-CATGCCTTGATCCACCTGTTC-3′; reverse: 5′-TGCAATGCATAGGGAATGATG-3′). The PCR program was executed as follows: 95 °C for 10 min; 3 cycles of 94 °C for 30 s, 64 °C for 30 s, and 72 °C for 45 s; 5 cycles of 94 °C for 30 s, 62 °C for 30 s, and 72 °C for 45 s; 10 cycles of 94 °C for 30 s, 60 °C for 30 s, and 72 °C for 45 s; followed by 17 cycles of 94 °C for 30 s, 58 °C for 30 s, and 72 °C for 45 s; with a final extension step of 72 °C for 5 min. The PCR products were purified using magnetic bead-based purification. Subsequently, a pre-sequencing PCR was conducted (96 °C for 1 min 30 s, 28 cycles of 96 °C for 15 s, 50 °C for 6 s, and 60 °C for 3 min 30 s). Following purification, the products were analyzed by capillary electrophoresis sequencing on the 3130XL Genetic Analyzer (Applied Biosystems, Foster City, CA, USA). Following the identification of the reference sequence, the reference sequence and raw sequencing data were analyzed using the Mutation Surveyor software (SoftGenetics, State College, PA, USA).

## Results

**Figure 1:**
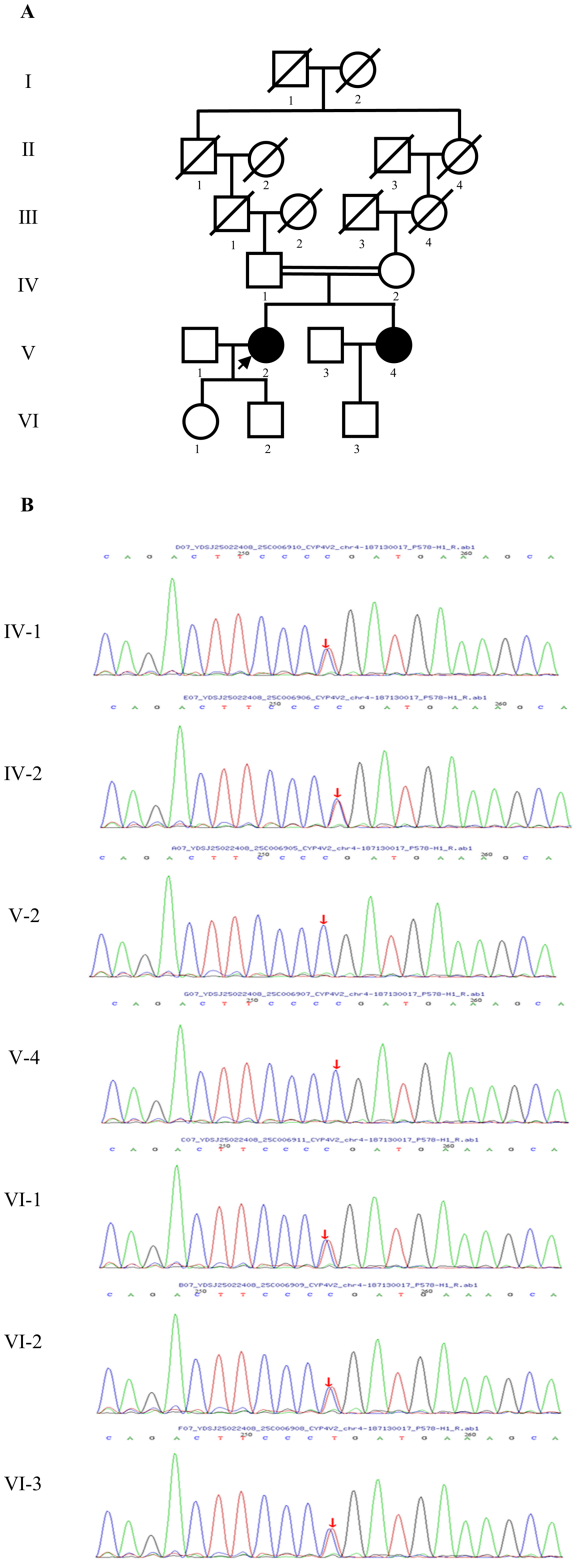
Pedigree of the Chinese family with Bietti crystalline dystrophy and Sanger sequencing. **(A)** IV-1 and IV-2 are second-degree paternal cousins. The arrow indicates the proband. Squares and circles, respectively, correspond to male and female. Empty and filled symbols are, respectively, used to distinguish unaffected individuals from those. **(B)** V-2 and V-4 were homozygous for the c.1091-2A > G mutation, while all other family members were heterozygous carriers.

A 46-year-old female proband (V-2) presented to the Affiliated Hospital of Nantong University with progressive bilateral visual impairment and was clinically suspected of having Bietti crystalline dystrophy (BCD). Her family, which included seven members—two affected individuals (V-2 and V-4) and five unaffected relatives (IV-1, IV-2, VI-1, VI-2, and VI-3)—was subsequently enrolled for clinical and genetic evaluation. Pedigree analysis revealed consanguinity due to a second-cousin marriage (Figure 1A), and the pattern of inheritance was consistent with autosomal recessive transmission.

The proband (V-2) first noticed vision loss at the age of 26, with marked progression over the past year. She also had a more than 10-year history of nyctalopia. In order to improve eyesight, she underwent cataract surgery in the right eye at our hospital. The proband’s sister (V-4) shared a similar clinical history. She experienced vision decline beginning at the age of 30 and the onset of nyctalopia over the past 2 years. No significant vision declined or nyctalopia (night blindness) was observed in other family members. The findings of the pedigree are summarized in Table 1.

**Table 1:** Clinical features and mutation of the pedigree with Bietti crystalline dystrophy.

Patient	Age	Sex	BCVA		Crystalline deposit	Fundus finding	c.1091-2A > G mutation
			OD	OS			
IV-1	70 y	Male	20/25	20/30	None	None	Heterozygous
IV-2	69 y	Female	20/30	20/30	None	None	Heterozygous
V-2	46 y	Female	HM	HM	Cornea, lens, retina	CD, CNV, CME pigmentation, RPE atrophy	Homozygous
V-4	42 y	Female	HM	20/200	Cornea, Lens, Retina	CD, CNV, MH, retinoschisis, pigmentation, RPE atrophy	Homozygous
VI-1	25 y	Female	20/20	20/20	None	None	Heterozygous
VI-2	17 y	Male	20/20	20/20	None	None	Heterozygous
VI-3	18y	male	20/20	20/20	None	None	Heterozygous

HM, hand motion; CD, crystalline deposits; CNV, choroidal neovascularization; CME, cystoid macular edema; MH, Macular hole; retinoschisis.

**Figure 2:**
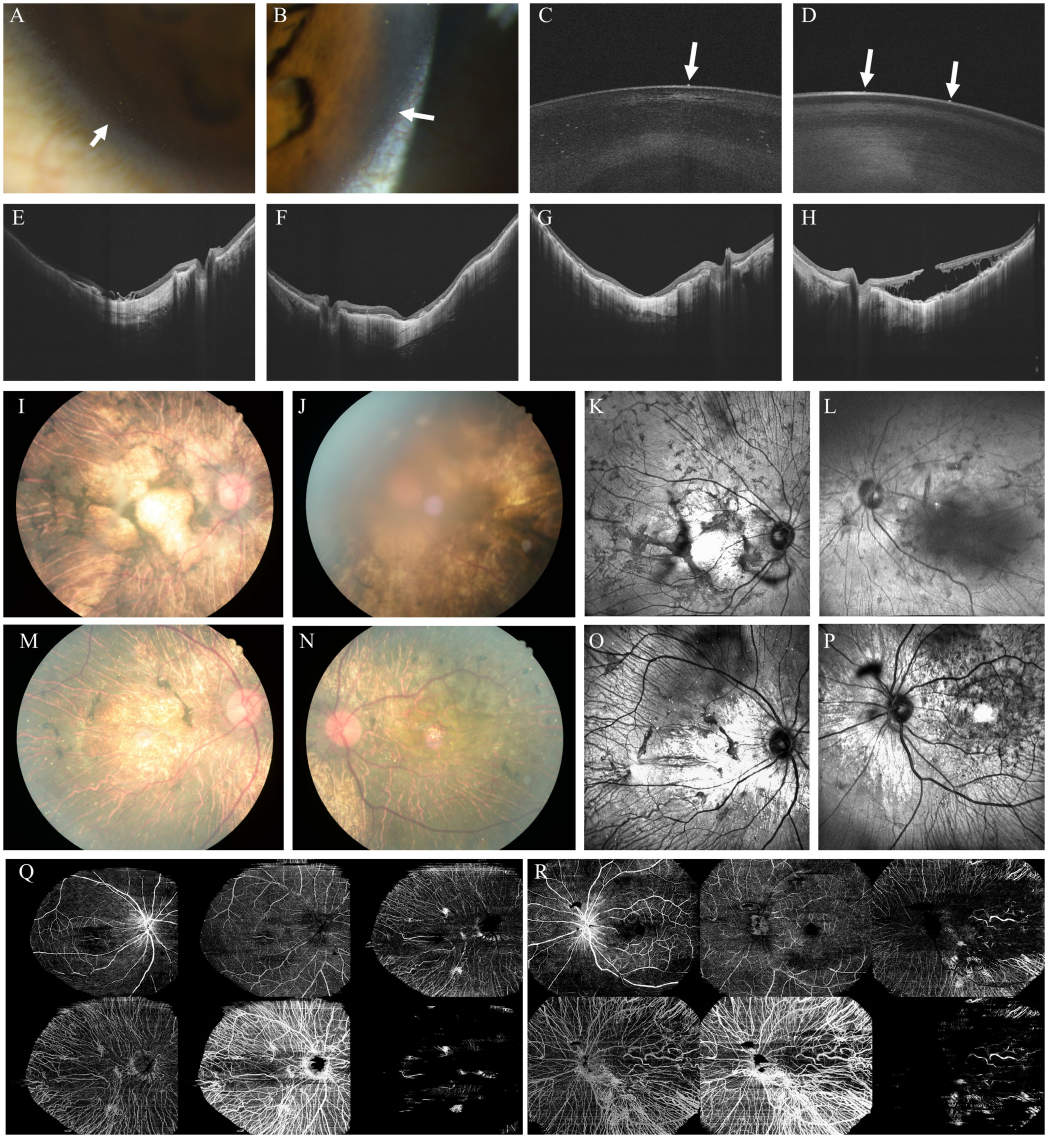
Multimodal anterior and posterior segment imaging of the proband and her sister. **(A,B)** Slit-lamp photographs of the anterior segment (**A**: proband; **B**: proband’s sister); **(C,D)** Anterior segment optical coherence tomography (AS-OCT) (**C**: proband; **D**: proband’s sister); **(E,F)** OCT of the proband (**O**: right eye; **P**: left eye); **(G,H)** OCT of the proband’s sister (**Q**: right eye; **R**: left eye); **(I,J)** Color fundus photographs of the proband (**E**: right eye; **F**: left eye); **(K,L)** NIR images of the proband (**K**: right eye; **L**: left eye); **(M,N)** Color fundus photographs of the proband’s sister (**I**: right eye; **J**: left eye); **(O,P)** NIR images of the proband’s sister (**K**: right eye; **L**: left eye). **(Q,R)** Optical coherence tomography angiography (OCTA) images of the proband’s sister showing the superficial capillary plexus (SCP), deep capillary plexus (DCP), avascular zone, choriocapillaris, choroid, and retinal pigment epithelium (RPE) layers (**M**: right eye; **N**: left eye).

Ophthalmic examinations of this family were as follows: Slit-lamp examination revealed multiple crystalline deposits at the corneal limbus in both the proband and her younger sister. Anterior segment optical coherence tomography (AS-OCT) showed hyperreflective foci anterior to and within the anterior lens capsule, as well as within the lens cortex, in both individuals, with more extensive involvement in the proband. Color fundus photography, near-infrared (NIR) imaging, and OCT demonstrated diffuse retinal pigment epithelium (RPE) atrophy accompanied by numerous crystalline deposits in both eyes of the proband and her sister. OCT angiography (OCTA) could not be performed in the proband due to poor visual acuity and unstable fixation, whereas successful imaging was achieved in her sister. Choroidal neovascularization (CNV) and outer retinal tubulation (ORT) were identified in both patients. In addition, the proband exhibited cystoid macular edema (CME), while her sister presented with a macular hole (MH) associated with retinoschisis. Comprehensive ophthalmic examinations of other family members revealed no significant abnormalities (Figure 2).

By NGS and targeted sequence capture array techniques, a homozygous mutation were found in the proband: c.1091-2A > G. The homozygous mutation had been reported in BCD patients as pathogenic mutations. Direct Sanger sequencing confirmed the presence of a homozygous variant in the proband and her sister, while heterozygous mutations were identified in other members of the pedigree (Figure 1). The segregation pattern of this homozygous variant was consistent with the clinical phenotype and genetic profile of the family, supporting its potential role as a disease-causing variant.

## Discussion

Yuzawa et al. (7) proposed a staging system for Bietti crystalline dystrophy (BCD), dividing the disease into three phases based on clinical severity and fundus appearance. Stage 1 (early phase) is characterized by preserved or slightly decreased visual acuity, focal RPE atrophy at the posterior pole, prominent intraretinal crystalline deposits within atrophic lesions, and peripheral pigmentary clumping. Stage 2 (intermediate phase) shows progressive visual loss, macular RPE–choriocapillaris atrophy extending beyond the posterior pole, and aggregation or whitening of crystalline deposits. Stage 3 (advanced phase) involves severe vision loss, diffuse RPE–choriocapillaris atrophy, bone spicule-like pigmentation, and a reduction in the quantity of visible crystalline deposits (8). These stages reflect the progressive retinal degeneration typical of BCD. In our family, however, the proband and her sister—despite being close in age and carrying the same homozygous CYP4V2 variant—exhibited markedly different structural phenotypes. This discordance suggests that the BCD staging system, while clinically useful, may not fully capture the phenotypic heterogeneity associated with the disease.

The diagnosis of BCD remains challenging, particularly in early stages. Limbal corneal crystalline deposits can be subtle and easily obscured by arcus senilis, and may go undetected without specialized imaging techniques such as confocal or specular microscopy (9, 10). Additionally, as RPE atrophy progresses, intraretinal crystalline deposits become less visible, and the appearance of bone spicule-like pigmentation can resemble that of retinitis pigmentosa (RP), often leading to misdiagnosis in advanced cases (11, 12). These factors may contribute to the underrecognition of BCD, especially in populations with limited access to genetic testing and high-resolution imaging.

In addition to the retinal and limbal corneal crystalline deposits commonly reported in BCD, crystal-like deposits may also occur in the lens. Chung et al. (13) described hyperreflective material on the inner surface of the anterior lens capsule, which gradually migrated toward the lens cortex. In our cases, both the proband and her sister exhibited hyperreflective deposits on the anterior and inner surfaces of the anterior lens capsule, with the proband showing more extensive crystalline accumulation. The origin of these lens deposits remains unclear. Potential sources include degenerated retinal cells, infiltrating inflammatory cells, and lens epithelial cells (14). This multilayered distribution suggests that different pathological processes may contribute to crystal formation. We propose that lens crystal deposition in BCD may result from the combined effects of three hypotheses: passive deposition of circulating crystal-like substances or metabolites, intrinsic degeneration of lens epithelial cells, and secondary changes associated with metabolic dysfunction. Rather than acting independently, these mechanisms may synergistically contribute to multi-site crystalline deposition. Unfortunately, the lens capsule and cortex from the proband were not preserved during cataract surgery, precluding further histological analysis. Future studies involving lens pathology may provide further insights into the underlying mechanisms.

Optical coherence tomography (OCT) revealed notable retinal structural abnormalities in both individuals, including diffuse retinal thinning, ellipsoid zone disruption, outer retinal tubulation (ORT), and choroidal neovascularization (CNV). The proband presented with pronounced cystoid macular edema (CME), whereas her sister exhibited a rare combination of a full-thickness macular hole (MH) and retinoschisis. While CNV, ORT, CME, and MH have been previously reported in BCD (15–22), retinoschisis has not been documented to date. Padhi et al. (23) described bilateral subfoveal neurosensory detachment, and Ji et al. (24) reported a full-thickness MH resembling our case. However, our patient exhibited more extensive schisis localized to the outer nuclear layer (ONL), representing a novel structural manifestation. Axial length measurements in both siblings were slightly above average. Interestingly, only the sister’s left eye, which had a relatively normal axial length (24.74 mm), developed MH and retinoschisis, while the longer right eye (25.59 mm) did not. The proband, despite modest axial elongation, showed CME but no MH or retinoschisis. These observations suggest that axial elongation alone does not fully account for macular structural abnormalities in BCD. We speculate that factors such as extensive RPE atrophy, microstructural fragility, vitreomacular traction, and local inflammation may play a more critical role in the pathogenesis of macular holes and retinoschisis. The asymmetry in phenotypic expression further supports the idea that additional modifying factors influence the severity and pattern of retinal degeneration in BCD.

Since multiple CYP4V2 mutations were first reported in 2004, increasing evidence has shown that BCD is linked to systemic disturbances in lipid metabolism (25). CYP4V2 encodes a microsomal *ω*-hydroxylase involved in processing omega-3 polyunsaturated fatty acids that support photoreceptor and RPE function, and the protein is expressed not only in retina and RPE but also in cornea and several systemic tissues such as kidney, liver, lung, and lymphocytes. Disruption of this metabolic pathway may impair retinal lipid turnover and alter PUFA-derived signaling molecules, contributing to crystalline deposition and progressive retinal degeneration in BCD (11). These molecular effects are consistent with the characteristic crystalline deposits observed across multiple retinal layers in our patients. The homozygous CYP4V2 splice-site mutation c.1091-2A > G identified in this family has been well established as a pathogenic variant in BCD. This variant is one of three most common CYP4V2 variants in Chinese population (26). This mutation disrupts normal mRNA splicing, simultaneously leading to the production of aberrant transcripts, skipping of exon 9, and markedly reduced CYP4V2 enzymatic activity. It is predicted to cause a major conformational change in CYP4V2 protein, which may result in a more severe disease phenotype compared with homozygous or heterozygous missense mutations (27). Moreover, chronic disruption of lipid turnover and secondary structural weakening of the retina may predispose patients to tractional changes, potentially contributing to the unusual phenotype of macular hole combined with retinoschisis observed. Together, these findings support the notion that the pathogenic splice-site defect in CYP4V2 triggers a cascade of metabolic dysfunction and structural instability that can explain the clinical manifestations in this family.

Although the proband and her sister carried the same homozygous splice-site mutation (c.1091-2A > G) in CYP4V2, their corneal and retinal phenotypes differed markedly. As a splice-site variant, c.1091-2A > G primarily disrupts pre-mRNA processing rather than directly altering the amino acid sequence. Importantly, splicing is often leaky rather than completely abolished, allowing a variable proportion of correctly spliced transcripts to be generated and translated into functional CYP4V2 protein. This variability in residual splicing efficiency may lead to differences in functional protein levels between individuals and thereby contribute directly to disease severity (28). In addition, genetic modifiers, epigenetic regulation, environmental exposures, age, metabolic status, and comorbid conditions may further modulate disease expression. Together, these factors likely underlie the intrafamilial phenotypic heterogeneity observed in this family and reflect the complexity of genotype–phenotype correlations in BCD.

Among the modifying influences that affect splicing fidelity and ultimately shape the disease phenotype, genetic factors are generally considered to exert the predominant effect, whereas non-genetic contributors also play meaningful roles and frequently interact with the underlying genetic background. Because pre-mRNA splicing is a sequence-dependent process governed by a tightly regulated network of splicing factors, cis- and trans-acting variants, together with broader polygenic or pathway-level differences, can substantially influence baseline splicing efficiency and disease susceptibility. Non-genetic influences such as metabolic or oxidative stress, age-associated cellular decline, stochastic fluctuations in transcription and splicing, and dynamic epigenetic modifications further modulate disease expression across the lifespan (29). Taken together, the observed phenotype reflects the combined effects of the core pathogenic variant and modifier genetic factors, which define the baseline susceptibility and potential trajectory of the disease. Non-genetic influences, including environmental exposures, epigenetic regulation, and stochastic events, further modulate how this trajectory is realized, shaping the rate, timing, and severity of disease progression. Importantly, when more granular clinical variations are considered, such as differences in progression rates among individuals with the same genotype, variability in age of onset, or the presence of milder manifestations despite an identical mutation, non-genetic factors often become increasingly influential and may even predominate in specific contexts. These factors primarily shape the timing, speed, and extent of disease manifestations, rather than the inherent pathogenicity of the mutation.

To further investigate the intrafamilial phenotypic variability observed between the proband and her sister, several additional examinations could be considered in future studies. Functional validation of the splice-site mutation using minigene assays could clarify its impact on mRNA splicing and the proportion of correctly spliced transcripts. Whole-exome or whole-genome sequencing may help identify potential modifier genes or polygenic influences. Lipid metabolic profiling could assess differences in retinal lipid metabolism, while advanced multimodal imaging (such as OCT angiography, or fundus autofluorescence) may detect subtle structural changes. Functional retinal testing, including electroretinography, could further evaluate variations in retinal function. However, these investigations were not performed in the present study due to limitations in patient availability, sample material, and the scope of the current research. Nevertheless, these approaches may provide valuable insights in future studies aimed at elucidating the mechanisms underlying phenotypic heterogeneity in BCD.

## Conclusion

The phenotype of BCD shows marked variability, with differences observed even among individuals of similar age who harbor the same pathogenic mutation. Given the phenotypic variability and overlapping clinical features of BCD with other retinal dystrophies, we recommend the combined use of multimodal ophthalmic imaging alongside genetic testing to achieve a more accurate and definitive diagnosis in clinical practice. Such an integrative approach not only enhances diagnostic precision but also facilitates early detection and family screening, which is particularly important given the progressive nature and genetic basis of BCD. Therapeutic interventions for BCD are approaching clinical viability. We believe that, with the support of emerging gene-editing technologies and current research advances, BCD is moving toward clinical translation.

## Data Availability

The original contributions presented in the study are included in the article/supplementary material, further inquiries can be directed to the corresponding author.
